# Impact of the first wave of the COVID-19 pandemic on non-COVID inpatient care in southern Spain

**DOI:** 10.1038/s41598-023-28831-6

**Published:** 2023-01-30

**Authors:** Antonia Gasch-Illescas, Marta Calle-Serrano, Antonio J. Vallejo-Vaz, Juan M. Praena-Fernández, Jose A. Guerrero, Enrique J. Calderón, Marina Pollán, Francisco J. Medrano

**Affiliations:** 1 Prevention in Health and Longevity Centre, Institut Pasteur de Lille, 59000 Lille, France; 2 University of Seville, 41004 Seville, Spain; 3 Infectious and Immune System Diseases-Epidemiology and Public Health, Institute of Biomedicine of Seville (IBiS) (Virgen del Rocío University Hospital, University of Seville, Junta de Andalucía, and Spanish Scientific Research Council-CSIC), 41013 Seville, Spain; 4 Internal Medicine Service, Virgen del Rocío University Hospital, 41013 Seville, Spain; 5 Clinical Epidemiology and Vascular Risk, Institute of Biomedicine of Seville (IBiS) (Virgen del Rocío University Hospital, University of Seville, Junta de Andalucía, and Spanish Scientific Research Council-CSIC), 41013 Seville, Spain; 6 Department of Statistics and Operations Research, University of Granada, Granada, Spain; 7 Clinical Documentation Service, Virgen del Rocío University Hospital, 41013 Seville, Spain; 8 Institute of Biomedicine of Seville (IBiS) (Virgen del Rocío University Hospital, University of Seville, Junta de Andalucía, and Spanish Scientific Research Council-CSIC), 41013 Seville, Spain; 9 Centro de Investigación Biomédica en Red de Epidemiología y Salud Pública (CIBERSP), 28029 Madrid, Spain; 10 National Center for Epidemiology, Instituto de Salud Carlos III ES, 28029 Madrid, Spain; 11 Present Address: Department of Medicine, Faculty of Medicine, University of Seville, 41013 Seville, Spain

**Keywords:** Health care, Epidemiology

## Abstract

We assessed the impact of the first wave of COVID-19 pandemic on non-COVID hospital admissions, non-COVID mortality, factors associated with non-COVID mortality, and changes in the profile of non-COVID patients admitted to hospital. We used the Spanish Minimum Basic Data Set with diagnosis grouped according to the Diagnostic Related Groups. A total of 10,594 patients (3% COVID-19; 97% non-COVID) hospitalised during the first wave in 2020 (27-February/07-June) were compared with those hospitalised within the same dates of 2017–2019 (average annual admissions: 14,037). We found a decrease in non-COVID medical (22%) and surgical (33%) hospitalisations and a 25.7% increase in hospital mortality among non-COVID patients during the first pandemic wave compared to pre-pandemic years. During the officially declared sub-period of excess mortality in the area (17-March/20-April, in-hospital non-COVID mortality was even higher (58.7% higher than the pre-pandemic years). Non-COVID patients hospitalised during the first pandemic wave (compared to pre-pandemic years) were older, more frequently men, with longer hospital stay and increased disease severity. Hospitalisation during the first pandemic wave in 2020, compared to hospitalisation during the pre-pandemic years, was an independent risk factor for non-COVID mortality (HR 1.30, 95% CI 1.07–1.57, p = 0.008), reflecting the negative impact of the pandemic on hospitalised patients.

## Introduction

The causal agent of the COVID-19 is a virus of the family *Coronaviridae* named severe acute respiratory syndrome coronavirus 2 (SARS-CoV-2). Its genetic sequence was published in February 2020^1^. Since the beginning of the pandemic in December 2019, SARS-CoV-2 has been spreading widely worldwide, resulting in more than 566 million confirmed cases and over 6.3 million deaths worldwide until July 2022, according to WHO^2^.

Mortality statistics are the most widely used source of information to compare health data across communities, regions or nations, and are basic to public health strategies^3^. Excess mortality is estimated by comparison between all-cause mortality and expected mortality, based on the risks of mortality (pre-COVID-19 period) in the population^4^. Spain has the highest European record of excess of mortality in 2020, followed by Italy, Switzerland, and France^5^. This excess of mortality cannot be only explained by the lethality of the virus on more or less vulnerable patients (direct effects). A part of deaths may be attributed to the collateral effects of the pandemic and the associated burden posed on healthcare systems and delivery of care; i.e.: overload of healthcare systems, limited access to emergency services, impairment of disease monitoring and of preventive medicine, or psychological and behavioural effects issued from the social confinement. This indirect mortality is partly balanced by the decrease of some usual external risks such as road and professional accidents during the COVID-19 confinement.

Most published studies on COVID-19 are focused on direct effects of the disease. Its indirect impact, such as excess mortality due to other causes than COVID-19 or effects of social restrictions on health outcomes remains insufficiently evaluated, in-hospital as well as out-hospital environment. While there are several studies exploring non-COVID-19 excess mortality during the pandemic, studies specifically assessing excess mortality overall and grouped by disease conditions in non-COVID hospitalised individuals are, however, rather limited^6–8^. Additionally, the profile of non-COVID patients admitted to hospital during the first wave are scarcely explored so far^7–11^.

The first case of COVID-19 in Andalusia was reported in Seville on February 26, 2020. The Spanish government implemented the first lockdown on March 15, 2020, including the mandate that all non-essential workers remain at home; this first lockdown was in place until June 21, 2020. Formal restrictions regarding access to health care were not implemented at regional level by the authorities. However, a contingency plan was implemented at the VRUH as of March 16, including: prioritization of teleworking for health-care workers when presence was not essential, in primary and hospital care; prioritization of external consultations via telephone and telemedicine, in primary and hospital care; cancellation of non-urgent face-to-face consultations, in primary and hospital care; postponement of scheduled and elective surgeries, except for oncological surgery and other conditions with risk of serious clinical deterioration; maintenance of urgent surgery; reduction of major ambulatory surgery operations to 50%; suspension of paediatric and adult kidney transplantation; maintenance of code 0 liver and heart transplantations; suspension of the non-heart beating donation program. These measures were maintained until the end of May 2020, the date when the de-escalation began progressively.

This paper aims to contribute to a better understanding of the high excess mortality observed during the first wave of COVID-19 in the south of Spain in spring 2020. This real-world data analysis is mainly focused on admissions, mortality, factors related to non-COVID mortality and changes in profile of non-COVID patients from a large hospital area of Andalusia. Variations in the number, type and severity of diagnoses contributing to hospitalizations and in-hospital mortality during the first wave of the COVID-19 pandemic (Feb–Jun 2020) were investigated and compared to the same period during the last 3-year pre-pandemic period. Results are discussed with in order to support future public health strategies, care planning and resource management in hospitals for the management of pandemic or analogous crises.

## Results

The study setting and population are described in detail in the “Methods” section. Briefly, the study was conducted at the Virgen del Rocío University Hospital (VRUH) (Seville, Andalusia; Spain), a large, 1500-bed tertiary-care university hospital that serves a population of more than 550,000 inhabitants. During the period of study, a total of 52,383 non-COVID patients (10,271 in 2020, and 42,112 in the 2017–19 period [13,788 in 2017; 14,271 in 2018; and 14,053 in 2019]) were admitted to the Hospital and included in the study.

Anonymous patient records were extracted and analysed from the MBDS database.

### Hospital admissions

Sub-period 1 (corresponding to the local pandemic first wave: February 27 to June 7, 2020)^12^: in 2020 10,594 individuals were admitted to hospital (323 with COVID-19 and 10,271 without COVID); the number of admissions for years 2017–19 were 42,112 (average per year: 14,037 ± 242) (Table 1). The number of non-COVID admissions in 2020 decreased by 26.8% compared with the average figure per year in the previous three pre-pandemic years (p < 0.001) (Table 1).

**Table 1:** Characteristics of non-COVID patients admitted to the Virgen del Rocío University Hospital between February 27 and June 7, 2020 (sub-period 1) and between March 17 and April 20, 2020 (sub-period 2), compared with the same sub-periods in 2017–19. *SD* standard deviation, *ICU* intensive care unit, *APR-DRG* all patient refined diagnosis related groups, *LOS* length of stay; *Chi-Square test; **Student T-test; ***Mann–Whitney U-test.

	Sub-period 1 (first COVID-19 pandemic wave in Seville)			Sub-period 2 (excess mortality in Andalusia)		
	2020	2017–19	p value	2020	2017–19	p value
Total admissions, n	10,271	42,112		2900	14,281	
Annual admissions, n	10,271	14,037	< 0.001*	2900	4760	< 0.001*
Sex (male), n (%)	4768 (46.4)	19,994 (47.4)	0.083*	1278 (44.1)	6765 (47.4)	0.001**
Age, mean ± SD	50.76 ± 4.9	49 ± 25.22	< 0.001**	50.99 ± 24.60	49.22 ± 25.22	0.001**
Emergency admissions, n (%)	6248 (60.8)	22,884 (54.3)	< 0.001*	1935 (66.7)	8094 (56.7)	< 0.001*
Days of hospitalisation, mean ± SD	7.09 ± 11.09	6.62 ± 10.6	< 0.001***	7.45 ± 12.31	6.67 ± 10.13	< 0.001***
Days of hospitalisation in ICU, mean ± SD	0.67 ± 4.96	0.65 ± 4.37	0.657**	0.67 ± 4.93	0.66 ± 4.23	0.900**
Medical APR DRG, n (%)	6229 (60.6)	23,876 (56.7)	< 0.001**	1852 (63.9)	8269 (57.9)	< 0.001**
Severity level, mean ± SD	1.82 ± 0.84	1.71 ± 0.8	< 0.001***	1.86 ± 0.86	1.72 ± 0.8	< 0.001**
Mortality level, mean ± SD	1.54 ± 0.79	1.45 ± 0.74	< 0.001***	1.58 ± 0.81	1.47 ± 0.75	< 0.001***
APR-DRG weigh, mean ± SD	0.89 ± 1.02	1.17 ± 1.33	< 0.001***	0.89 ± 1.09	1.18 ± 1.39	< 0.001***
APR-DRG-adjusted LOS index, mean ± SD	0.93 ± 0.87	0.94 ± 0.98	0.052***	0.94 ± 1.01	0.94 ± 0.95	0.976***
Deaths at hospital, n (%)	507 (4.94)	552.0 (3.93)	< 0.001**	186 (6.41)	192.3 (4.04)	< 0.001*

Significant values are in bold.

Sub-period 2 (corresponding to the identified period of excess mortality in Andalusia according to the national monitoring report on daily mortality: March 17 to April 20, 2020)^13^: there were 3190 hospital admissions in 2020 (290 with COVID-19 and 2900 without COVID) and 14,281 in 2017–19 (average per year: 4760 ± 158) (Table 1), that is, a decrease of 39.1% in the number of admissions in 2020 (p < 0.001) (Table 1).

### Characteristics of hospitalized patients

The characteristics of non-COVID patients hospitalized in sub-periods 1 and 2 during 2020 and during the period 2017–19 are shown in Table 1.

During sub-period 1, the mean age of patients hospitalized in 2020 was higher than in the previous three years (50.76 versus 49.00 years, p < 0.001).

Emergency admissions increased by 12.0% in 2020 compared with the period 2017–19 (60.8% versus 54.3%, p < 0.001). An increased in the length of hospitalization was also observed in 2020 (mean 7.09 days, versus 6.62 days in 2017–19, p < 0.001). The percentage of medical diagnoses increased by 6.9% in 2020 compared with the previous years (60.6% versus 56.7%, p < 0.001), and they were associated with a lower level of complexity according to the mean APR weight (0.89 in 2020, versus 1.17 in 2017–19, p < 0.001) and higher levels of severity (1.82 versus 1.71, respectively, p < 0.001) and mortality (1.54 versus 1.45, p < 0.001).

The same patterns were observed between year 2020 versus years 2017–19 for the sub-period 2, with similar or even stronger differences in these variables between year 2020 and years 2017–19 (Table 1).

### Mortality

**Figure 1:**
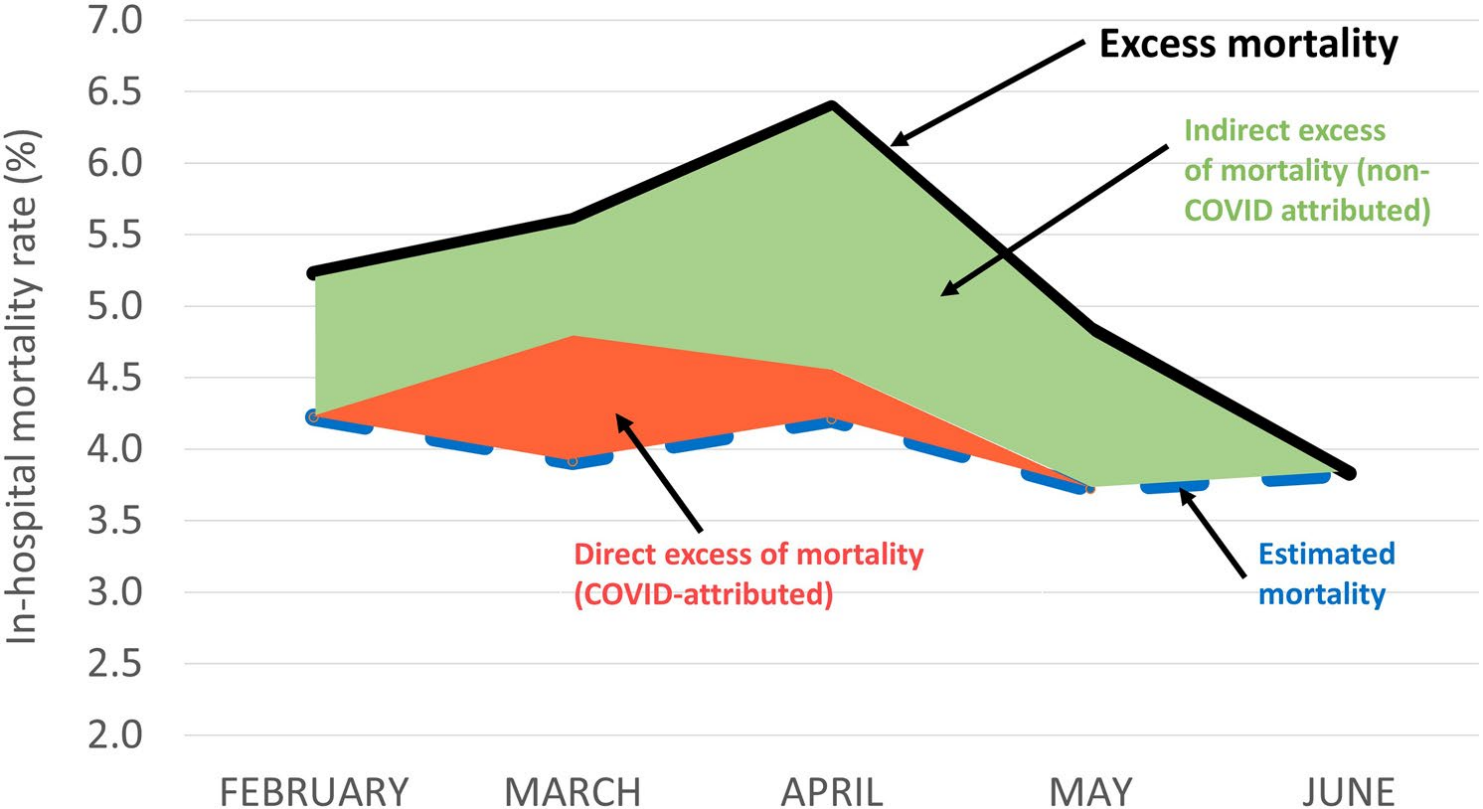
Estimated and observed trends of in-hospital mortality at the Virgen del Rocío University Hospital during the first pandemic wave (sub-period 1). Estimated mortality is the in-hospital mortality for this period observed in 2017–2019. Excess mortality due to COVID-19 results in the addition of both direct (COVID-attributed mortality) and indirect (non-COVID attributed mortality). Mortality rate is expressed as deaths for 100 admissions.

During sub-period 1, global in-hospital mortality in 2020 was 5.31 per 100 admissions (17.33% for patients diagnosed with COVID-19, and 4.94% for mortality not related to COVID). The corresponding in-hospital mortality (not related to COVID) in years 2017–19 for the same sub-period 1 ranged between 3.71 per 100 admissions in 2017 to 4.24 per 100 in 2019 (average of 3.93 ± 0.28 per 100 admissions), Table 2. Therefore, there was a 25.6% increase (p < 0.001) in in-hospital mortality from non-COVID causes in year 2020 compared with the prior 2017–19 years. The estimated and observed trends of in-hospital mortality during sub-period 1 are shown in Fig. 1.

**Table 2:** In-hospital and global mortality at the Virgen del Rocío health area between February 27 and June 7, 2020 (sub-period 1: first COVID-19 pandemic wave in Seville), compared with the same period in 2017–19. *Chi square test.

	2020	2019	2018	2017	p value*
Admissions in VRHU	10,594	14,053	14,271	13,788	
Non-COVID	10,271	14,053	14,271	13,788	
COVID	323	0	0	0	
In-hospital deaths	563	596	549	511	
Non-COVID	507	596	549	511	
COVID	56	0	0	0	
In-hospital mortality per 100 admissions	5.31	4.24	3.85	3.71	**< 0.001**
Non-COVID	4.94	4.24	3.85	3.71	**< 0.001**
COVID	17.33	0	0	0	
Reference population of VRHU health area	480,346	478,950	480,743	478,042	
Total deaths in the VRHU health area	1160	1105	1045	1016	
Global mortality rate per 100.000 habitants	241.49	230.71	217.37	212.53	

Significant values are in bold.

During sub-period 2, in-hospital mortality in 2020 remained similar for patients diagnosed with COVID-19 (17.3 per 100 admissions) and slightly increased for non-COVID-related mortality causes (6.41 per 100 admissions) when compared with sub-period 1 in the same year 2020 (overall in-hospital mortality during sub-period 2 in year 2020: 5.8 per 100 admissions). The overall in-hospital mortality (not related to COVID-19) for sub-period 2 in years 2017–19 was 4.04 per 100 admissions on average. Therefore, a 58.7% increase (p < 0.001) in non-COVID-related mortality was observed for this sub-period 2 in 2020 compared with years 2017–19.

A total of 1160 patients died during sub-period 1 in 2020 in the VRUH health area, which represents a mortality rate of 241.5 per 100,000 inhabitants; this figure was higher than the ones registered in years 2017–19, which ranged between 212.5 and 230.7 per 100,000 inhabitants (for the same VRUH area) (Table 2). The average mortality for the sub-period 1 in 2017–19 was 219.9 per 100,000 inhabitants; these results indicate an excess mortality of 10% in 2020 compared to the earlier years studied.

**Figure 2:**
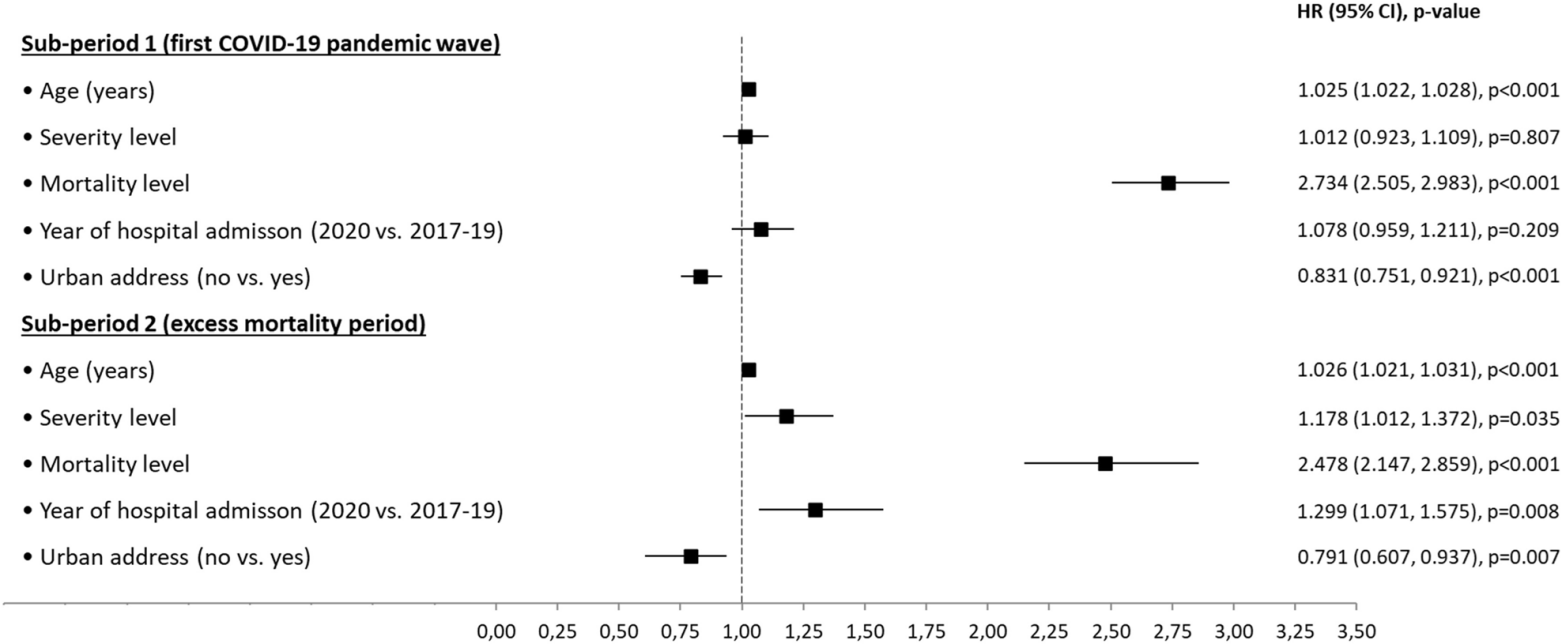
Impact of different variables on in-hospital mortality in patients without COVID-19 admitted to the Virgen del Rocío University Hospital. Multivariable Cox analysis with adjusted hazard ratios for the sub-period 1 (February 27 to June 7, 2020) and the sub-period 2 (March 17 to April 20, 2020). HR, hazard ratio; CI, confidence interval.

Multivariable Cox regression analyses for the impact of different variables on in-hospital mortality in patients without COVID for sub-periods 1 and 2 are shown in Fig. 2. In sub-period 1, the adjusted risk of in-hospital death increased with the higher mortality level (HR 2.73 per level, 95% CI 2.50–2.98) and older age (HR 1.02 per year, 95% CI 1.02–1.03), whereas this risk decreased for those not living in urban areas (HR 0.83 for rural versus urban areas, 95% CI 0.75–0.92). In sub-period 2, in addition to the previous variables (with similar magnitudes of risks), the following variables were also found to increase the risk of in-hospital mortality: a higher severity level (HR 1.18 per level, 95% CI 1.01–1.37) and year of hospital admission (HR 1.30 for year 2020 versus years 2017–19, 95% CI 1.07–1.57).

### Changes in APR-DRGs profiles

The characteristics of non-COVID patients admitted to hospital for the 40 most frequent APR-DRGs between the sub-period 1 in 2020, compared with the same sub-period in years 2017–19, are summarized in Supplementary Tables 1 and 2 for medical and surgical admissions, respectively. Overall, both for medical and surgical admissions, mean age, days of hospitalisation, severity of diagnoses and in-hospital mortality were higher in 2020 compared to the 2017–19 period. No difference was found in the APR-DRG-adjusted LOS index between pandemic and pre-pandemic periods for both medical and surgical admissions. In-hospital non-COVID mortality increased by 19.3% for medical disorders (6.8% versus 5.7%, p < 0.001) and by 33.3% for surgical procedures (2.0% versus 1.5%, p = 0.026).

Regarding medical diseases, the number of admissions was significantly lower in 23 (57.5%) of the 40 most frequent APR-DRGs, whereas admissions increased only in four APR-DRGs (lymphoma, myeloma and non-acute leukaemia; bipolar disorders; kidney and urinary tract infections; and false labour). Variations in mean age, length of hospital stay, mean severity, APR-DRGs-adjusted LOS index and mortality for medical procedures are described in Supplementary Table 1.

The medical APR-DRGs categories most impacted by a decrease in the number of hospitalisations (greater than 50%) were the following: digestive disorders, therapeutics (other chemotherapy); cardiac disorders, nervous system disorders and soft tissues disorders. Overall, the total medical admissions decreased 22% in the 2020 study period versus the pre-pandemic period.

Regarding surgical procedures, the number of hospital admissions in 2020 was significantly lower in 24 (60%) of the 40 most frequent APR-DRGs, whereas the number of admissions increased only in one APR-DRGs (Supplementary Table 2). Variations in mean age, length of hospital stay, mean severity, APR-DRGs-adjusted LOS index and mortality for surgical procedures are described in Supplementary Table 2.

Surgical procedures most impacted by a decrease in hospital admissions (greater than 50%) were namely: kidney transplant, anal and perineal procedures, trauma procedures (knee joint replacement; other significant HIP and femur surgery; foot and toe procedures); hernia procedures (inguinal, femoral and umbilical hernia procedures; hernia procedures except inguinal, femoral and umbilical), otorhinolaryngology procedures (tonsil and adenoid procedures), breast procedures except mastectomy, and percutaneous cardiac intervention without acute myocardial infarction. Total surgical procedures decreased by 33% in the 2020 study period versus the pre-pandemic period.

## Discussion

The present study reports, for the first time, the overall impact of the COVID-19 pandemic on the health care provided and outcomes in a large tertiary university hospital beyond the effect directly related to the SARS-CoV-2 disease itself, i.e. among patients without COVID. In this study, the number of admissions, demographic and clinical characteristics were analysed, and mortality among hospitalized patients during this first pandemic wave were compared to patients admitted during the same period within the previous three years. This analysis has been carried out for two sub-periods, i.e. a broader one (sub-period 1), which includes the entire first pandemic wave in the studied area^12^, and a shorter one (sub-period 2), which corresponds only to the period of time when the excess of mortality was officially recorded in the first wave^13^.

During sub-period 1, 12,950 confirmed cases of COVID-19 were reported in the region of Andalusia, of which 2456 cases corresponded to Seville, representing a cumulative incidence of 153/100,000 and 128/100,000 people in Andalusia and Seville, respectively^12^. Therefore, the estimated seroprevalence of SARS-CoV-2 in Spain^14^ in the first pandemic wave was 3.4% [2.3–4.9] and 2.7% [1.4–4.8] in Seville and Andalusia respectively (subjects resulting positive either on print-of-care test or immunoassay), i.e., 66,000 cases in Seville and 227,000 in Andalusia up to May 11th, 2020. In the health area evaluated, which covers a population of about 550,000 inhabitants, the officially reported incidence of COVID-19 during the first pandemic wave was low with a maximum 14-day cumulative incidence rate of 74.1 confirmed SARSCoV-2 cases per 100,000 individuals^12^, with a total of 323 admissions and 56 deaths due to COVID-19 during the first sub-period of analysis.

The most relevant findings of this study are the decrease in the number of hospital admissions, the change in the profile of the patients admitted to hospital, and the increase of the in-hospital and overall mortality of patients without COVID-19.

Firstly, the number of hospital admissions decreased by 26.8% during the first sub-period of the pandemic wave in 2020. This decrease was even larger in the second sub-period (39.1%). This reduction was observed both in patients with medical and with surgical conditions, with a reduction of admissions in 23 of the 40 most frequent medical diseases and in 24 of the 40 surgical conditions. These results are consistent with those previously reported in several hospital-based studies^15–21^. Moreover, emergency admissions increased by 12% in 2020 compared to the 2017–19 period. This result could be at least partly explained by the fact that patients seeking emergency care may have presented with conditions at a more advanced stage upon arrival to the hospital; this is supported by the higher level of disease severity in 2020 for the two periods analysed (Table 1). The self-dissuasion of patients from seeking care earlier, given the fear generated by the pandemic, together with the limited access to primary care services, are factors likely contributing to the delay of care attention.

Secondly, the clinical and demographic profile of non-COVID patients admitted during the first pandemic wave was different from those patients admitted in the years before. During 2020, compared with the period 2017–19, patients admitted were older and the proportion of men was higher. As well, the number of urgent admissions and the severity of the patient status increased, both overall and stratified by medical and surgical conditions, and the length of stay was longer. Similar changes in some of these variables have been reported in other studies analysing specific conditions as myocardial infarction^22,23^, stroke^23,24^, cancer^25,26^, chronic pulmonary obstructive disease^27^ or emergency surgical treatment^28^.

Albeit the patients admitted in the pandemic sub-periods had a more severe status than those admitted in the prior years, no significant change in the use of intensive care beds for non-COVID patients was observed between the pandemic period and the prior years. This observation may be influenced by the use of intensive care beds by COVID-19 patients, however the rate of use of stays was maintained.

A possible conclusion is that, despite the care overload during the pandemic, health care professionals, both in primary care and in hospitals, with the support of the community (e.g., by complying with public health recommendations), kept health care as optimized as possible within the ongoing circumstances at that time, minimising the impact on hospitals.

To date, changes in the characteristics of patients admitted during the pandemic have only been described for some specific medical and surgical diseases. A study carried out in the United States comparing the first wave of the 2020 pandemic with the previous years, observed that patients with symptoms of myocardial infarction, stroke or asthma delayed seeking care due to the COVID-19 pandemic^29^.

The available studies that have been conducted at a national scale have shown a reduction in overall admissions, without signaling any increase on specific conditions. However, in the VRUH in Seville, the number of admissions related to “lymphoma, myeloma and non-acute leukaemia”, “false labour”, “kidney and urinary tract infections” and “bipolar disorders”, increased in the first pandemic wave (Supplementary Table 1). Although the authorities did not decide on a specific strategy for these diseases, they did not impose restrictions for malignancy disorders nor for conditions with risk of short-term clinical deterioration.

The significant increase in admissions related to onco-haematological conditions could be explained by the fact that the number of patients treated on an outpatient basis was drastically reduced and, consequently, these patients were admitted to the hospital to receive treatment. The increased number of available hospital beds due to the reduction on admissions for non-malignancy and serious conditions may have led to a higher rate of hospitalizations of prioritized diseases related to nephro-urology and obstetrical services, even at early stages of the diseases, partly related to the reduced accessibility to outpatient clinics and treatments. This hypothesis is supported by the unchanged in-hospital mortality for these conditions (Supplementary Table 1). The increase of admissions for bipolar disorders supports the findings from previous studies observing a high prevalence of mental disorders in the global population during the first pandemic wave, including depression, anxiety, distress and insomnia^30^ and especially bipolar disorders^31^. Our findings are also consistent with a study carried out at the Helio Hospital in Germany, where the increase in admissions concerned quite different diseases (myocardial infarction, intestinal obstruction, pneumonitis, pulmonary embolism)^10^. The interpretation proposed here is that this heterogeneity results from uncoordinated local managements of care services in a period of crisis, for which health services and hospitals were not prepared and equipped and did not have a defined strategy; this situation affected differently to each care service and hospital, as did also differ the strategy and contingency plans put in place in each case; additionally, the severity and impact of the pandemic varied from one region to another; all together, therefore, necessarily led to differences from one hospital to another.

This hypothesis should be tested by comparing similar data from other hospitals in Spain and other countries, and should be interpreted in the context of the management of the hospital itself during this period.

At last, during the first pandemic wave of COVID-19, an excess of in-hospital mortality has been found in patients without COVID-19, which was 25.6% for the first sub-period of analysis (p < 0.001), and 58.7% for the second one (p < 0.001).

We expected to find in 2020 patients having more severe conditions and longer delays to seek or receive care and therefore higher mortality than in pre-pandemic period. Multivariable analyses (Fig. 2) partially confirmed this hypothesis and showed a higher risk of in-hospital mortality for urban versus rural address, for patients with higher mortality level, and for older patients. The identification of admission during 2020 as an independent risk factor for mortality reflects the strong impact of the pandemic on the non-COVID in-hospital mortality, especially during the second sub-period of analysis.

**Figure 3:**
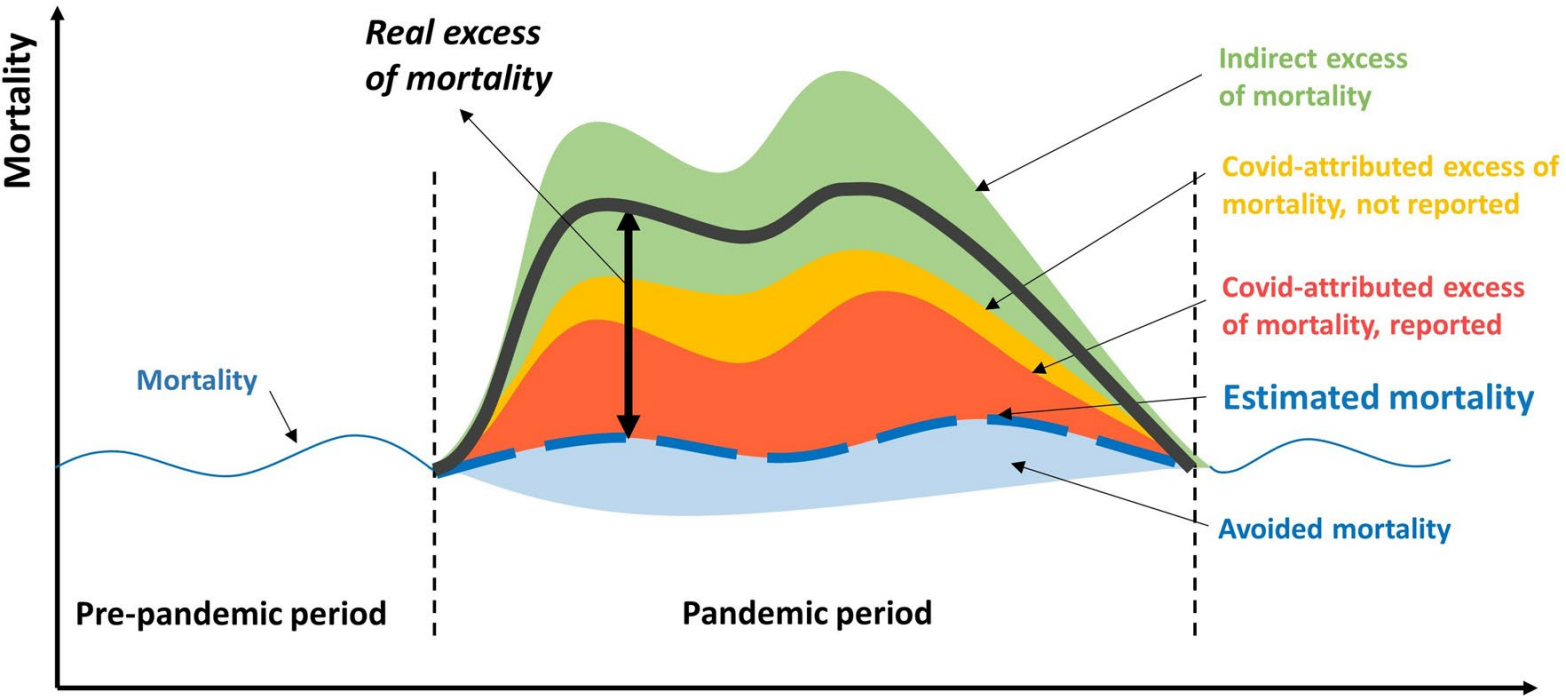
Theoretical representation of components of excess mortality in the COVID-19 pandemic. Excess mortality due to COVID-19 results in the addition of both direct (reported and not reported COVID-attributed deaths) and indirect mortality (due to the indirect effects of the pandemic on the health care system and on people’s behaviour), and the subtraction of avoided deaths (due to social restrictions).

The data on overall mortality in the reference population for the study area confirm the existence of an increase in the number of patients who died outside the hospital regardless of the period considered. This excess of mortality, beyond deaths reported in the international mortality registration system, may be attributed to a delay in reporting and to attribution errors (e.g. deaths from other respiratory diseases or other non-respiratory causes) that reflect the complications by COVID-19. These findings are congruent with previous studies suggesting that national registries underestimated the COVID-19 mortality rate in the first weeks of the pandemic^32,33^. Actually, as summarized in Fig. 3, the full impact of the pandemic might be much stronger than that indicated by the COVID-19 death reports, both inside and outside the hospital^34^.

The increase in the number of patients who died out-of-hospital could be related to health and non-health factors, including the health system overload, the suspension of non-urgent interventions and face-to-face consultations, the fear of patients to go to hospitals, and the impact of lockdown and other restrictive measures. Our findings on mortality are globally congruent with those of a study carried out with data from 24 European countries in which mortality was analysed in the period from March to June 2020 and compared with those that occurred in 2016–2019^5^.

However, there are large disparities on the reported in-hospital mortality among countries during the pandemic. For instance, studies carried out in Italy and US^6,8^ reported an increase of in-hospital mortality. In the United Kingdom^7^ it was estimated that one "preventable death" (non-COVID) occurred for every 30 hospitalised COVID patients. In Germany^11^, relatively few individuals were affected by COVID during the first wave; and Romania^9^ did not report any significant excess mortality during the same period.

The present study has several limitations, related mainly to the use of administrative electronic health records (EHR) as the source of information. The MBDS is an administrative database that must be completed by all hospitals in Spain. The clinical variables were obtained through the hospital discharge reports and, as such, there could be some missing information and variability issues. However, the coding is carried out by experts who received specific training and it is conducted using a standardized coding regulation^35^, which reduces the probability of information bias. Secondly, the analysis was restricted to data related to one hospital only. Nevertheless, this is a large study that includes the analysis of all hospitalized patients in the largest hospital of the autonomous region of Andalusia.

The comparison of the pandemic period is made with the findings observed in the previous three years in order to minimize the possible effect of factors affecting a particular year and so to reduce the risk of bias. All relevant variables available have been included in the study.

In conclusion, the present study shows for the first time a complete picture of the impact of the pandemic in a large hospital during the first pandemic wave, through a comparison with pre-pandemic years. A sharp decrease of non-COVID medical and surgical hospitalizations (22% and 33% respectively) was observed, together with changes in the characteristics of patients admitted including an increase in the age of the patients, a longer length of stay, and a higher severity of the condition. Similarly, an increase of 25.6% in in-hospital mortality, and an excess of overall mortality rate of 10.6%, were observed during this period.

Our results contribute to identify weaknesses in the health care system under crisis situations, and provide important elements to improve strategies for optimizing the response of the health system to similar challenges. Management strategies on health care and the robustness of the health systems are determinant on the mortality and of delivery of health care in non-COVID patients.

## Methods

### Setting

The study was conducted at the Virgen del Rocio University Hospital (VRUH), a 1500-bed tertiary-care university hospital in Seville, Spain, which provides approximately the 10% of acute care inpatient bed capacity in the region of Andalusia, Spain. The hospital serves a population of more than 550,000 inhabitants, which may be referred for outpatient and inpatient care in all medical areas (internal medicine and specialties, surgery, orthopaedics and traumatology, emergency and critical care, mental health, paediatrics, and obstetrics/gynaecology). In 2019, it recorded 48,765 hospital admissions, 42,854 surgical interventions and 330,142 admissions at the emergency department^36^. An analysis of real-world data was conducted from Minimum Basic Data Set (MBDS) registries.

### Study periods

Two periods were considered for the present study: a 130-day period corresponding to the local pandemic wave (subperiod 1: February 27 to June 7, 2020)^12^, and a 33-day period included within the latter, corresponding to the identified period of excess mortality in Andalusia according to the national monitoring report on daily mortality (subperiod 2: March 17 to April 20, 2020)^13^. No official data on excess mortality was available specifically for Seville. For the region of Andalusia, official excess mortality was 21.7%. The two periods considered in our study were selected in order to test the hypothesis that the period of excess mortality may also be the period with the largest impact on inpatient care. For each subperiod, data from patients admitted to VRUH for any cause were collected and compared with patients admitted within the same dates in years 2017, 2018 and 2019.

### Collection of data and studied variables

Data from hospitalized patients were obtained from the (MBDS), a standard management tool in the Spanish National Health System for the coding of hospital admissions. The diagnoses at hospital discharge were coded according to the Tenth Version of the International Classification of Disease (ICD-10-EN ^35^, and grouped according to the refined version (APR, "All Patient Refined") of the Diagnostic Related Groups (DRG) of the primary admission diagnosis in version 36.0^37^, which allows a more refined classification of hospital cases by providing information on the severity of the disease and the risk of mortality.

Data included demographic characteristics (age, sex, municipality of residence), variables related to the episode of hospitalisation (number of admissions during the study period, type of admission, length of stay (LOS), days spent in the intensive care unit (ICU) if applicable, severity and mortality indexes according to APR-DRG definitions, Spanish weight value of each APR DRG, Spanish APR-DRG-adjusted LOS index and in-hospital mortality), diagnoses at hospital discharge, and in-hospital or in the community cause of death (if applicable).

The population served by the VRUH was considered for each calendar year. The overall mortality data of the population served by the VRUH were obtained from the Statistical Service of the City of Seville (for the basic metropolitan health areas) and the Institute of Statistics and Cartography of Andalusia (for non-metropolitan basic health areas). Data from both sources come directly from the computerized civil registers of the Spanish Ministry of Justice.

The study was approved by the Andalusian Biomedical Research Ethics Committee, VRUH, Seville, Spain. All methods were performed in accordance with the guidelines of the WMA Declaration of Helsinki. Patient records were extracted from the MBDS database in an anonymised way. Patient data were provided anonymised for analyses.

### Data analysis

A descriptive, uni- and bivariate analysis of the variables was carried out. Between-group comparisons were conducted using independent-samples T-test or Mann–Whitney U-test for normally and not normally distributed variables, respectively. Categorical variables and proportions were compared using Chi-squared test (or Fisher Exact Test where appropriate.

Cox regression was carried out to assess any potential association between the risk of death and demographic and clinical variables in our study. The model allows evaluating, within a set of variables, which ones have an influence on the risk function, controlling for possible confounders. Variables included in the final model were selected using the conditional backward elimination method. The model provides an estimate of risk (hazard ratio and 95% confidence intervals) that accounts for the time at risk of each individual. The analysis time was the period between the date of hospital admission and date of discharge (or death for patients who died in the hospital).

In-hospital mortality rates and community mortality rates were estimated. The statistical software IBM SPSS (IBM Corporation, Somers, NY, USA), version 26.0, was used for the analyses. Tests were two-sided; statistical significance was set as p < 0.05.

## Supplementary Information

Supplementary Table 1.

Supplementary Table 2.

## Data Availability

The datasets used and analysed during the current study are available from the corresponding author on reasonable request.

## Abbreviations

APR: All patient refined
MBDS: Minimum basic data set
COPD: Chronic obstructive pulmonary disease
DRG: Diagnosis related groups
EHR: Electronic health record
HR: Hazard ratio
ICD-10-EN: Tenth version of the International Classification of Diseases
ICU: Intensive care unit
LOS: Length of stay
VRUH: Virgen del Rocio University Hospital

## References

[1] Loeffelholz MJ, Tang YW (2020). Laboratory diagnosis of emerging human corona virus infections-the state of the art. Emerg. Microbes Infect..

[2] World Health Organization. WHO Coronavirus Disease (COVID-19) Dashboard. https://covid19.who.int (continuously updated).

[3] Ochoa-Sangrador C (2021). Impact of COVID-19 on mortality in the autonomous community of Castilla y León. Gac. Sanit..

[4] Banerjee A (2020). Estimating excess 1-year mortality associated with the COVID-19 pandemic according to underlying conditions and age: a population-based cohort study. Lancet.

[5] Vestergaard LS, Nielsen J, Richter L, Schmid D, Bustos N (2020). Excess all-cause mortality during the COVID-19 pandemic in Europe—preliminary pooled estimates from the EuroMOMO network, March to April 2020. Euro Surveill..

[6] Bartolomeo N, Giotta M, Trerotoli P (2021). In-hospital mortality in non-COVID-19-related diseases before and during the pandemic: A regional retrospective study. Int. J. Environ. Res. Public Health..

[7] Fetzer, T. & Rauh, C. *Quantifying Excess Mortality Among Non COVID-19 Patients in Healthcare Settings*. (Cambridge Working Papers in Economics 2252, Faculty of Economics, University of Cambridge, 2022).

[8] Dang A, Thakker R, Li S, Hommel E, Mehta HB, Goodwin JS (2022). Hospitalizations and mortality from non-SARS-CoV-2 causes among medicare beneficiaries at US hospitals during the SARS-CoV-2 pandemic. JAMA Netw. Open..

[9] Vladescu C, Ciutan M, Rafila A (2022). In-hospital admissions and deaths in the context of the COVID19 pandemic, in Romania. GERMS.

[10] Bollmann A (2021). Helios hospitals, Germany. Hospitalisations for emergency-sensitive conditions in Germany during the COVID-19 pandemic: insights from the German-wide Helios hospital network. Emerg. Med. J..

[11] König S (2022). A comparative analysis of in-hospital mortality per disease groups in Germany before and during the COVID-19 pandemic from 2016 to 2020. JAMA Netw. Open..

[12] Servicio de Estadísticas Sanitarias. Consejería de Salud y Familias de la Junta de Andalucía. Consejería Informe COVID-19 en Andalucía. Situación de COVID-19 según fecha de diagnóstico por provincias. https://www.juntadeandalucia.es/institutodeestadisticaycartografia/badea/operaciones/consulta/anual/39409?CodOper=b3_2314&codConsulta=39409 (continuously updated).

[13] Instituto de Salud Carlos III. Vigilancia de los excesos de mortalidad por todas las causas, MoMo(ISCIII). https://www.isciii.es/QueHacemos/Servicios/VigilanciaSaludPublicaRENAVE/EnfermedadesTransmisibles/MoMo/Documents/informesMoMo2021/MoMo_Situacion%20a%2011%20de%20mayo_CNE.pdf (2021).

[14] Pollán M (2020). Prevalence of SARS-CoV-2 in Spain (ENE-COVID): A nationwide, populationbased seroepidemiological study. Lancet.

[15] Quevedo-Ramirez A, Al-Kassab-Córdova A, Mendez-Guerra C, Cornejo-Venegas G, Alva-Chavez KP (2020). Altitude and excess mortality during COVID-19 pandemic in Peru. Respir. Physiol. Neurobiol..

[16] Butt AA (2020). Hospital admission rates, length of stay, and in-hospital mortality for common acute care conditions in COVID-19 versus pre-COVID-19 era. Public Health.

[17] Caminiti C (2021). Effects of the COVID-19 epidemic on hospital admissions for non-communicable diseases in a large Italian University-Hospital: A descriptive case-series study. J. Clin. Med..

[18] Moynihan R (2021). Impact of COVID-19 pandemic on utilisation of healthcare services: A systematic review. BMJ Open.

[19] Rennert-May E (2021). The impact of COVID-19 on hospital admissions and emergency department visits: A population-based study. PLoS ONE.

[20] Abebe W (2021). Trends of follow-up clinic visits and admissions three-months before and during COVID-19 pandemic at Tikur Anbessa specialized hospital, Addis Ababa, Ethiopia: An interrupted time series analysis. BMC Health Serv. Res..

[21] Cai Y (2021). Impact of the COVID-19 pandemic on a tertiary care public hospital in Singapore: Resources and economic costs. J. Hosp. Infect..

[22] Mafham MM (2020). COVID-19 pandemic and admission rates for and management of acute coronary syndromes in England. Lancet.

[23] Kiss P, Carcel C, Hockham C, Peters SAE (2021). The impact of the COVID-19 pandemic on the care and management of patients with acute cardiovascular disease: a systematic review. Eur. Heart. J. Qual. Care Clin. Outcomes.

[24] Nogueira RG (2021). Global impact of COVID-19 on stroke care and IV thrombolysis. Neurology.

[25] Ranganathan P (2021). Impact of COVID-19 on cancer care in India: A cohort study. Lancet Oncol..

[26] Elkrief A (2022). Learning through a pandemic: The current state of knowledge on COVID-19 and cancer. Cancer Discov..

[27] Chan KPF (2020). Significant reduction in hospital admissions for acute exacerbation of chronic obstructive pulmonary disease in Hong Kong during coronavirus disease 2019 pandemic. Respir. Med..

[28] Winter Beatty J (2021). Impact of the COVID-19 pandemic on emergency adult surgical patients and surgical services: An international multi-center cohort study and department survey. Ann. Surg..

[29] Rivera R, Rosenbaum JE, Quispe W (2020). Excess mortality in the United States during the first three months of the COVID-19 pandemic. Epidemiol. Infect..

[30] Wu T (2021). Prevalence of mental health problems during the COVID-19 pandemic: A systematic review and meta-analysis. J. Affect. Disord..

[31] Abbas MJ (2020). The early impact of the COVID-19 pandemic on acute care mental health services. Psychiatr. Serv. Mar..

[32] Woolf SH, Chapman DA, Sabo RT, Weinberger DM, Hill L (2020). Excess deaths from COVID-19 and other causes, March–April 2020. JAMA.

[33] Hubert H, Baert V, Beuscart JB (2020). Use of out-of-hospital cardiac arrest registries to assess COVID-19 home mortality. BMC Med. Res. Methodol..

[34] COVID-19 Excess Mortality Collaborators. Estimating excess mortality due to the COVID-19 pandemic: A systematic analysis of COVID-19-related mortality, 2020–21. *Lancet* **399**, 1513–1536. 10.1016/S0140-6736(21)02796-3 (2022). 10.1016/S0140-6736(21)02796-3 PMC8912932 35279232

[35] Pastor, M. D, Navalon, R. & Pato, S. (eds). Subdirección General de Información Sanitaria e Innovación. Ministerio de Sanidad, Servicios Sociales e Igualdad. Manual de Codificación CIE-10-ES Diagnósticos, 2016. https://www.sanidad.gob.es/estadEstudios/estadisticas/normalizacion/CIE10/CIE10ES_2016_norm_Manual_codificacion_Diagnosticos.pdf (2016).

[36] Memorias del Hospital Universitario Virgen del Rocío. https://www.hospitaluvrocio.es/entrada-blog/memoria/ (accessed 28 Mar 2022).

[37] Registro de Atención Sanitaria Especializada RAE CMBD del Ministerio de Sanidad. Manual de definiciones y glosario de términos. Spanish. https://pestadistico.inteligenciadegestion.mscbs.es/publicoSNS/D/rae-cmbd/raecmbd/glosario-de-terminos-y-definiciones/glosario-de-terminos-y-definiciones (2021).

