# Supplementary material for: Impact of the first wave of the COVID-19 pandemic on non-COVID inpatient care in southern Spain
Source: Sci Rep. 2023 Jan 30;13:1634. doi: 10.1038/s41598-023-28831-6 (PMC9885064; doi:10.1038/s41598-023-28831-6)
Supplement: Supplementary file 1 — Supplementary Table 1. [file 41598_2023_28831_MOESM1_ESM.pdf]

CODE	APR DRG	Incomes				Age in year (mean)			Days of hospitalisation (mean)			Severity (mean)			APR IEMA raw (mean)			In-hospital mortality (%)		
		2020 n	2017-19 mean	% change	p*	2020	2017-19	p**	2020	2017-19	p**	2020	2017-19	p**	2020	2017-19	p**	2020	2017-19	p
044	INTRACRANIAL HEMORRHAGE	54	64	-16	0.393	69.09	66.74	0.351	7.07	9.17	0.093	2.26	2.35	0.542	0.73	0.86	0.223	38.90	30.10	0.218*
045	CVA AND PRECEREBRAL OCCLUSION WITH INFARCTION	121	129	-6	0.910	70.27	70.22	0.973	6.53	6.69	0.795	2.16	2.02	0.065	0.69	0.75	0.419	17.50	7.00	**0.001***
053	SEIZURE	74	125	-41	**<0.001**	20.12	13.40	**0.025**	4.46	4.26	0.781	1.82	1.67	0.137	0.72	0.77	0.461	5.40	0.30	**0.003***
058	OTHER DISORDERS OF NERVOUS SYSTEM	41	93	-56	**<0.001**	44.61	42.92	0.693	3.90	5.05	0.392	1.85	1.69	0.188	0.52	0.64	0.353	7.30	1.10	**0.030¶**
113	INFECTIONS OF UPPER RESPIRATORY TRACT	44	79	-44	**<0.001**	30.16	30.66	0.913	5.64	4.16	**0.041**	1.59	1.66	0.558	1.29	0.89	**0.023**	0.00	1.70	1.000*
115	OTHER EAR, NOSE, MOUTH, THROAT AND CRANIAL OR FACIAL DIAGNOSES	91	114	-20	**<0.001**	41.37	40.31	0.724	3.46	2.62	0.041	1.34	1.34	0.994	0.97	0.77	**0.048**	0.00	1.50	0.589¶
133	RESPIRATORY FAILURE	109	101	+8	**<0.001**	66.49	61.57	0.071	7.95	8.17	0.828	2.59	2.60	0.850	0.87	0.89	0.876	32.10	29.90	0.672*
136	RESPIRATORY MALIGNANCY	55	93	-41	**0.003**	65.58	64.25	0.551	6.45	8.54	**0.010**	2.42	2.29	0.212	0.71	0.89	**0.043**	27.30	30.50	0.637*
139	OTHER PNEUMONIA	131	162	-19	**0.002**	60.05	59.54	0.852	6.70	6.62	0.870	2.05	2.13	0.306	0.87	0.81	0.395	8.40	8.00	0.890*
140	CHRONIC OBSTRUCTIVE PULMONARY DISEASE	95	93	+2	0.033	71.83	71.14	0.660	7.56	6.30	0.149	2.56	2.31	**0.004**	0.89	0.76	0.191	11.60	8.20	0.324*
143	OTHER RESPIRATORY DIAGNOSES EXCEPT SIGNS, SYMPTOMS AND MISCELLANEOU	54	54	0	0.338	42.31	42.01	0.947	4.54	5.30	0.353	1.74	1.84	0.474	0.68	0.75	0.471	7.40	3.70	0.272¶
144	RESPIRATORY SIGNS, SYMPTOMS AND MISCELLANEOUS DIAGNOSES	92	105	-12	**0.040**	55.58	39.58	**<0.001**	5.30	5.52	0.717	2.07	1.76	**0.001**	0.68	0.58	0.110	5.40	2.20	0.110*
190	ACUTE MYOCARDIAL INFARCTION	45	54	-17	0.317	73.84	72.82	0.656	5.18	7.59	**0.001**	2.04	2.13	0.545	0.89	1.23	**0.002**	6.70	18.50	0.065¶
191	CARDIAC CATHETERIZATION FOR CORONARY ARTERY DISEASE	48	78	-38	**<0.001**	65.48	66.05	0.765	4.13	3.80	0.592	1.42	1.45	0.711	0.96	0.83	0.322	0.00	0.00	NA
192	CARDIAC CATHETERIZATION FOR OTHER NON-CORONARY CONDITIONS	75	198	-62	**<0.001**	58.33	57.94	0.873	4.64	4.15	0.630	1.72	1.56	**0.052**	0.84	0.70	0.186	1.30	0.70	0.450*
194	HEART FAILURE	284	360	-21	**<0.001**	77.02	75.90	0.181	6.91	7.76	**0.034**	2.31	2.26	0.306	0.82	0.89	0.119	17.30	11.20	**0.006***
198	ANGINA PECTORIS AND CORONARY ATHEROSCLEROSIS	42	60	-30	**0.020**	76.55	73.54	0.130	0.48	0.49	**<0.001**	2.10	1.86	**0.045**	0.75	1.07	**< 0.001**	4.80	6.60	1.000*
240	DIGESTIVE MALIGNANCY	55	66	-17	0.340	66.45	66.64	0.947	8.07	9.52	0.331	2.33	2.42	0.431	0.73	0.90	0.147	23.60	35.20	0.106*
243	OTHER ESOPHAGEAL DISORDERS	32	124	-74	**<0.001**	16.66	10.37	0.195	2.19	1.71	0.393	1.53	1.25	**0.030**	0.58	0.47	0.339	0.00	0.00	NA
249	OTHER GASTROENTERITIS, NAUSEA AND VOMITING	60	79	-24	**0.013**	40.47	33.26	0.118	6.67	5.37	0.257	2.03	1.85	0.137	1.00	0.93	0.529	5.00	4.60	1.000*
254	OTHER DIGESTIVE SYSTEM DIAGNOSES	84	119	-29	**0.004**	46.10	43.07	0.411	7.17	4.94	0.146	1.85	1.52	**0.002**	1.10	0.82	**0.050**	4.80	1.70	0.103¶
281	MALIGNANCY OF HEPATOBILIARY SYSTEM AND PANCREAS	89	78	+14	0.171	64.46	66.75	0.203	7.51	9.30	0.095	2.31	2.43	0.225	0.77	0.90	0.180	23.60	25.10	0.778*
282	DISORDERS OF PANCREAS EXCEPT MALIGNANCY	83	102	-19	0.244	58.16	62.77	**0.05**	9.88	8.84	0.296	1.77	1.79	0.801	1.23	1.01	0.090	1.20	3.90	0.315¶
283	OTHER DISORDERS OF THE LIVER	34	84	-59	**<0.001**	51.65	58.91	0.121	7.65	4.21	**0.018**	2.03	1.96	0.571	0.94	0.55	**0.032**	0.00	3.60	0.606¶
351	OTHER MUSCULOSKELETAL SYSTEM AND CONNECTIVE TISSUE DIAGNOSES	52	74	-29	0.129	42.65	44.73	0.606	3.98	3.04	0.157	1.42	1.34	0.380	0.74	0.64	0.382	0.00	0.00	NA
385	OTHER SKIN, SUBCUTANEOUS TISSUE AND BREAST DISORDERS	41	85	-52	**<0.001**	47.37	49.70	0.565	4.93	2.75	**0.001**	1.46	1.21	**0.018**	1.16	0.75	0.109	2.40	0.40	0.257*
463	KIDNEY AND URINARY TRACT INFECTIONS	121	104	+17	**0.003**	47.03	43.05	0.254	6.31	5.94	0.411	1.98	1.88	0.212	0.84	0.87	0.278	4.10	2.60	0.394*
466	MALFUNCTION, REACTION, COMPLICATION OF GENITOURINARY DEVICE OR PROCE	101	89	+14	0.509	57.29	57.17	0.369	6.96	5.77	0.135	2.87	2.69	**0.010**	0.84	0.78	0.495	2.90	1.10	0.355*
468	OTHER KIDNEY AND URINARY TRACT DIAGNOSES, SIGNS AND SYMPTOMS	71	88	-19	**<0.001**	55.08	48.33	**0.047**	2.56	3.02	0.264	1.58	1.73	0.110	0.61	0.65	0.674	4.20	0.80	0.067¶
469	ACUTE KIDNEY INJURY	95	94	+1	0.101	67.02	69.22	0.292	9.19	9.70	0.615	2.13	2.19	0.444	1.06	1.03	0.766	12.60	11.30	0.728*
560	VAGINAL DELIVERY	935	1069	-13	**<0.001**	30.69	31.06	0.112	2.95	3.11	**0.002**	1.47	1.42	**0.021**	1.12	1.20	**<0.001**	0.00	0.00	NA
565	FALSE LABOR	57	49	+16	**0.048**	31.18	28.37	**0.003**	2.88	1.61	0.220	1.25	1.27	0.739	1.31	0.72	0.243	0.00	0.00	NA
566	ANTEPARTUM WITHOUT O.R. PROCEDURE	136	188	-28	**0.006**	31.27	30.01	**0.037**	2.66	2.60	0.829	1.47	1.51	0.536	0.76	0.77	0.943	0.00	0.00	NA
640	NEONATE BIRTH WEIGHT > 2499 GRAMS, NORMAL NEWBORN OR NEONATE WITH O	47	66	-29	0.067	0.00	0.00	NA	6.36	7.61	0.354	1.77	1.77	0.946	1.49	1.33	0.486	0.00	0.00	NA
660	MAJOR HEMATOLOGIC OR IMMUNOLOGIC DIAGNOSES EXCEPT SICKLE CELL CRISIS	58	85	-32	**0.001**	37.17	33.25	0.348	9.14	8.23	0.426	2.31	2.09	**0.05**	0.94	1.00	0.624	1.70	3.10	1.000*
691	LYMPHOMA, MYELOMA AND NON-ACUTE LEUKEMIA	115	55	+110	**<0.001**	48.28	56.07	**0.005**	7.83	14.69	**<0.001**	1.73	2.08	**0.001**	0.74	1.25	**<0.001**	7.80	8.50	0.832*
696	OTHER CHEMOTHERAPY	67	217	-69	**<0.001**	39.24	30.83	**0.001**	4.60	4.53	0.828	1.99	2.02	0.549	1.01	0.91	0.360	0.00	0.20	1.000*
720	SEPTICEMIA AND DISSEMINATED INFECTIONS	98	127	-23	**0.002**	71.05	68.85	0.281	10.88	11.74	0.487	3.11	2.90	**0.022**	0.94	1.05	0.262	25.50	33.70	0.122*
753	BIPOLAR DISORDERS	73	44	+67	**<0.001**	45.21	45.58	0.864	12.82	16.80	**0.028**	1.48	1.53	0.550	0.76	0.99	**0.025**	0.00	0.00	NA
861	SIGNS, SYMPTOMS AND OTHER FACTORS INFLUENCING HEALTH STATUS	61	85	-28	**<0.001**	44.97	41.88	0.412	6.59	5.22	0.243	1.89	1.50	**0.043**	0.89	0.79	0.538	11.50	12.90	0.757*
	**TOTAL**	6229	7959	**-22**	**<0.001**	48.86	46.67	**0.001**	6.78	6.26	**0.031*****	1.92	1.83	**<0.001*****	0.91	0.92	0.857	6.80	5.70	**0.001***

* Chi-Square test; ** Student T-test; *** Mann-Whitney U-test; ¶ Fisher Exact Test; NA, not applicable

Highlighted in orange when values in 2020 are significantly higher compared to 2017-19

Highlighted in blue when values in 2020 are significantly lower compared to 2017-19

Supplementary Table 1 Characteristics of non-COVID patients admitted for the 40 most frequent medical APR DRGs to the Virgen del Rocío University Hospital between February 27 and June 7, 2020 (first COVID-19 pandemic wave in Seville), compared with the same period in 2017-2019
