# Supplementary material for: Impact of the first wave of the COVID-19 pandemic on non-COVID inpatient care in southern Spain
Source: Sci Rep. 2023 Jan 30;13:1634. doi: 10.1038/s41598-023-28831-6 (PMC9885064; doi:10.1038/s41598-023-28831-6)
Supplement: Supplementary file 2 — Supplementary Table 2. [file 41598_2023_28831_MOESM2_ESM.pdf]

CODE	APR DRG	Incomes				Age in year (mean)			Days of hospitalisation (mean)			Severity (mean)			APR IEMA raw (mean)			In-hospital mortality (%)		
		2020 n	2017-19 mean	% change	*p**	2020	2017-19	*p***	2020	2017-19	*p***	2020	2017-19	*p***	2020	2017-19	*p***	2020	2017-19	*p*
021	OPEN CRANIOTOMY EXCEPT TRAUMA|01|P	148	163	**-9**	0.367	46.58	49.48	0.199	13.78	14.70	0.779	1.91	1.78	0.143	0.75	0.85	0.592	4.10	3.90	0.938*
024	OPEN EXTRACRANIAL VASCULAR PROCEDURES	159	150	**+6**	**< 0.001**	66.74	67.83	0.407	5.19	6.08	0.183	2.48	2.32	0.058	0.63	0.68	0.427	3.10	4.40	0.478*
097	TONSIL AND ADENOID PROCEDURES	22	68	**-67**	**<0.001**	20.50	11.82	0.092	1.36	1.47	0.674	1.18	1.10	0.479	0.95	0.80	0.332	0.00	0.00	NA
098	OTHER EAR, NOSE, MOUTH AND THROAT PROCEDURES	109	186	**-41**	**< 0.001**	49.84	42.32	**0.001**	4.36	3.75	0.329	1.38	1.26	0.054	1.11	0.97	0.268	0.00	0.00	NA
120	MAJOR RESPIRATORY AND CHEST PROCEDURES	42	40	**+4**	0.906	63.02	57.23	0.060	4.45	7.00	**0.002**	1.55	1.60	0.650	0.63	0.90	**0.004**	2.40	0.80	0.450¶
121	OTHER RESPIRATORY AND CHEST PROCEDURES	38	52	**-27**	0.183	52.47	50.75	0.690	3.34	6.72	**<0.001**	1.55	1.55	0.968	1.17	1.62	**0.002**	0.00	0.60	1.000¶
163	CARDIAC VALVE PROCEDURES WITHOUT AMI OR COMPLEX PRINCIPAL DIAGNOSIS	58	66	**-13**	**0.001**	60.88	63.44	0.312	17.33	16.16	0.560	2.21	2.13	0.522	1.13	1.05	0.413	1.70	5.00	0.465¶
171	PERMANENT CARDIAC PACEMAKER IMPLANT WITHOUT AMI, HEART FAILURE OR S	89	108	**-18**	0.113	77.51	75.66	0.207	4.97	4.76	0.754	1.71	1.57	0.084	0.89	0.95	0.566	2.20	0.00	**0.046***
174	PERCUTANEOUS CARDIAC INTERVENTION WITH AMI	129	123	**+5**	0.339	63.44	63.97	0.671	4.41	6.37	**<0.001**	1.60	1.59	0.812	0.87	1.10	**<0.001**	1.60	1.70	1.000¶
175	PERCUTANEOUS CARDIAC INTERVENTION WITHOUT AMI	133	271	**-51**	**< 0.001**	62.81	63.04	0.897	4.98	5.58	0.330	1.64	1.85	**0.010**	1.15	1.14	0.931	0.80	0.60	0.583¶
226	ANAL AND PERINEAL PROCEDURES	17	72	**-76**	**< 0.001**	49.00	49.59	0.891	2.88	3.38	0.581	1.12	1.19	0.498	1.29	1.33	0.890	0.00	0.50	1.000*
227	HERNIA PROCEDURES EXCEPT INGUINAL, FEMORAL AND UMBILICAL	26	97	**-73**	**< 0.001**	60.54	57.64	0.349	4.27	3.81	0.589	1.35	1.41	0.584	1.13	0.89	0.127	0.00	0.70	1.000*
228	INGUINAL, FEMORAL AND UMBILICAL HERNIA PROCEDURES	42	125	**-66**	**< 0.001**	52.6	56.22	0.353	3.14	1.75	**0.017**	1.36	1.27	0.373	1.36	0.86	**<0.001**	0.00	0.00	NA
230	MAJOR SMALL BOWEL PROCEDURES	75	89	**-15**	0.100	55.87	60.04	0.169	13.87	14.35	0.824	1.95	2.07	0.371	0.93	0.89	0.659	6.70	5.60	0.755*
231	MAJOR LARGE BOWEL PROCEDURES	112	99	**+14**	0.190	65.06	64.50	0.749	12.24	10.43	0.381	1.81	1.65	0.093	0.93	0.91	0.780	2.70	1.50	0.671¶
234	APPENDECTOMY WITHOUT COMPLEX PRINCIPAL DIAGNOSIS|	68	95	**-28**	**<0.001**	24.66	25.20	0.846	3.43	2.99	0.366	1.09	1.03	0.186	1.16	0.96	0.202	0.00	0.00	NA
260	MAJOR PANCREAS, LIVER AND SHUNT PROCEDURES	42	45	**-7**	**< 0.001**	62.29	60.68	0.533	17.21	14.16	0.347	2.12	2.06	0.696	1.21	1.02	0.328	0.00	2.20	1.000*
263	CHOLECYSTECTOMY	67	132	**-49**	**< 0.001**	60.25	57.07	0.132	4.10	2.76	**0.008**	1.49	1.40	0.363	1.27	0.86	**0.021**	1.50	0.40	0.346*
301	HIP JOINT REPLACEMENT	107	141	**-24**	0.063	75.87	70.71	**0.003**	6.80	6.11	0.080	1.39	1.32	0.270	0.94	0.82	**0.023**	5.60	1.90	**0.032***
302	KNEE JOINT REPLACEMENT	49	193	**-75**	**< 0.001**	70.12	69.80	0.821	4.41	4.53	0.839	1.16	1.21	0.487	1.03	0.93	0.142	0.00	0.20	1.000¶
305	AMPUTATION OF LOWER LIMB EXCEPT TOES	52	71	**-27**	0.089	71.85	67.80	**0.031**	71.85	67.80	0.865	9.13	9.36	0.813	0.63	0.63	0.993	7.70	3.30	0.234¶
308	HIP AND FEMUR FRACTURE REPAIR	122	132	**-8**	0.722	79.98	75.28	**0.003**	6.50	7.19	0.113	1.98	1.85	0.092	0.72	0.78	0.141	3.30	3.10	1.000¶
309	OTHER SIGNIFICANT HIP AND FEMUR SURGERY	24	62	**-61**	**< 0.001**	30.58	37.52	0.254	8.63	7.02	0.398	1.75	1.47	0.181	0.73	0.80	0.753	0.00	0.80	1.000*
310	VERTEBRAL AND INTERVERTEBRAL SPINAL PROCEDURES INCLUDING DISC PROCE	39	69	**-44**	**0.001**	49.77	48.79	0.744	6.56	4.69	0.249	1.41	1.38	0.187	0.99	0.69	0.067	0.00	0.00	NA
313	KNEE AND LOWER LEG PROCEDURES EXCEPT FOOT	100	139	**-28**	**0.028**	49.21	45.53	0.090	6.33	5.88	0.636	1.50	1.37	0.058	1.07	0.99	0.499	0.00	0.30	1.000*
314	FOOT AND TOE PROCEDURES	29	68	**-57**	**< 0.001**	45.86	49.62	0.339	3.72	4.25	0.597	1.48	1.50	0.855	0.85	0.89	0.828	0.00	0.00	NA
315	SHOULDER, UPPER ARM AND FOREARM PROCEDURES EXCEPT JOINT REPLACEMEN	94	124	**-24**	**< 0.001**	54.54	53.22	0.604	3.14	3.21	0.848	1.47	1.42	0.397	0.92	0.96	0.671	0.00	0.00	NA
320	OTHER MUSCULOSKELETAL SYSTEM AND CONNECTIVE TISSUE PROCEDURES	48	86	**-44**	**0.002**	55.79	42.95	**<0.001**	5.29	4.45	0.361	1.40	1.40	0.948	1.01	0.76	0.073	2.10	0.80	0.401¶
361	SKIN GRAFT FOR SKIN AND SUBCUTANEOUS TISSUE DIAGNOSES	47	76	**-38**	**0.021**	63.26	58.40	0.139	5.70	5.67	0.987	1.55	1.33	0.063	0.70	0.92	0.308	0.00	0.40	1.000¶
362	MASTECTOMY PROCEDURES	60	72	**-17**	0.648	53.92	52.56	0.471	4.35	4.29	0.930	1.17	1.20	0.575	1.17	1.15	0.721	0.00	0.00	NA
363	BREAST PROCEDURES EXCEPT MASTECTOMY	68	145	**-53**	**< 0.001**	58.12	53.74	**0.003**	2.15	2.87	**0.037**	1.18	1.24	0.229	0.85	0.99	0.155	0.00	0.00	NA
364	OTHER SKIN, SUBCUTANEOUS TISSUE AND RELATED PROCEDURES	51	49	**+5**	0.105	57.94	51.25	**0.021**	5.61	5.15	0.837	1.14	1.29	**0.041**	0.75	1.03	0.281	2.00	0.00	0.259¶
440	KIDNEY TRANSPLANT	12	60	**-80**	**< 0.001**	34.50	51.40	**0.025**	15.08	16.39	0.743	2.17	2.12	0.851	0.99	1.06	0.753	0.00	0.00	NA
442	KIDNEY AND URINARY TRACT PROCEDURES FOR MALIGNANCY	43	40	**+7**	0.201	61.65	62.45	0.865	5.21	5.98	0.180	1.58	1.48	0.405	0.86	0.96	0.296	2.30	0.80	0.457¶
443	KIDNEY AND URINARY TRACT PROCEDURES FOR NON-MALIGNANCY	75	89	**-15**	0.104	50.37	44.90	0.091	4.96	5.95	0.363	1.64	1.49	0.145	0.86	1.07	**0.039**	1.30	1.10	1.000*
446	URETHRAL AND TRANSURETHRAL PROCEDURES	89	158	**-44**	**< 0.001**	69.65	67.33	0.146	2.62	2.57	0.849	1.54	1.37	**0.015**	0.91	0.88	0.607	0.00	0.00	NA
513	UTERINE AND ADNEXA PROCEDURES FOR NON-MALIGNANCY EXCEPT LEIOMYOMA	55	99	**-44**	**< 0.001**	50.91	50.75	0.947	2.38	2.69	0.509	1.22	1.19	0.649	0.73	0.82	0.446	0.00	0.30	1.000*
540	CESAREAN SECTION WITHOUT STERILIZATION	377	346	**+9**	0.465	32.24	32.50	0.476	4.81	5.03	0.418	1.51	1.58	0.084	1.06	1.07	0.813	0.00	0.10	1.000¶
950	EXTENSIVE O.R. PROCEDURE UNRELATED TO PRINCIPAL DIAGNOSIS	57	86	**-33**	**0.021**	54.18	53.99	0.952	13.49	10.06	0.188	2.21	2.06	0.261	0.89	0.87	0.931	1.80	4.30	0.701¶
951	MODERATELY EXTENSIVE O.R. PROCEDURE UNRELATED TO PRINCIPAL DIAGNOSIS	48	83	**-42**	**< 0.001**	40.83	50.04	**0.017**	5.42	7.91	0.176	1.71	1.78	0.573	0.69	0.96	0.107	0.00	4.00	0.375¶
	**TOTAL**	5893	11470	**-33**	**<0.001**	54.76	52.77	**0.008**	8.65	6.97	**<0.001*****	1.77	1.58	**<0.001*****	0.98	0.94	0.101	2.00	1.50	**0.026***

* Chi-Square test; ** Student T-test; *** Mann-Whitney U-test; ¶ Fisher Exact Test; NA, not applicable

Highlighted in orange when values in 2020 are significantly higher compared to 2017-19

Highlighted in blue when values in 2020 are significantly lower compared to 2017-19

Supplementary Table 2 Characteristics of non-COVID patients admitted for the 40 most frequent surgical APR DRGs to the Virgen del Rocío University Hospital between February 27 and June 7, 2020 (first COVID-19 pandemic wave in Seville), compared with the same period in 2017-2019.
